# Patterns of Geographical and Potential Adaptive Divergence in the Genome of the Common Carp (*Cyprinus carpio*)

**DOI:** 10.3389/fgene.2019.00660

**Published:** 2019-07-12

**Authors:** Jian Xu, Yanliang Jiang, Zixia Zhao, Hanyuan Zhang, Wenzhu Peng, Jianxin Feng, Chuanju Dong, Baohua Chen, Ruyu Tai, Peng Xu

**Affiliations:** ^1^Key Laboratory of Aquatic Genomics, Ministry of Agriculture, CAFS Key Laboratory of Aquatic Genomics and Beijing Key Laboratory of Fishery Biotechnology, Chinese Academy of Fishery Sciences, Beijing, China; ^2^State Key Laboratory of Marine Environmental Science, College of Ocean and Earth Sciences, Xiamen University, Xiamen, China; ^3^Henan Academy of Fishery Science, Zhengzhou, China; ^4^College of Fishery, Henan Normal University, Xinxiang, China; ^5^Laboratory for Marine Biology and Biotechnology, Pilot National Laboratory for Marine Science and Technology, Qingdao, China

**Keywords:** common carp, population genomics, linkage disequilibrium, haplotype, selective sweep

## Abstract

The common carp, *Cyprinus carpio*, is a cyprinid fish species cultured in Europe and Asia. It accounts for >70% of freshwater aquaculture production worldwide. We conducted a population genomics analysis on *C. carpio* using high-throughput SNP genotyping of 2,198 individuals from 14 populations worldwide to determine the genetic architecture of common carp populations and the genetic bases for environmental adaptation. Structure analyses including phylogeny and principal component analysis were also conducted, showing distinct geographical patterns in European and Asian populations. The linkage disequilibrium block average lengths of the 14 populations ranged from 3.94 kb to 36.67 kb. Genes within selective sweep regions were identified by genome scanning among the different populations, including *gdf6a*, *bmpr1b*, and *opsin5*. Gene Ontology and KEGG enrichment analyses revealed potential trait-related loci and genes associated with body shape, scaling patterns, and skin color. This population genomics analysis may provide valuable clues for future genome-assisted breeding of *C. carpio*.

## Introduction

The common carp, *Cyprinus carpio*, is one of the most important cyprinid species due to its food value and complex paleotetraploidized genome. It is cultured in >100 countries, and the annual global production of *C carpio* is > 4.56 million metric tons. This is approximately 10% of the global freshwater aquaculture production (FAO Fisheries and Aquaculture Department; Bostock et al., 2010). Common carp provide high value protein as food and some strains, such as koi, are popular ornamental fish. Common carp have been cultured for several thousand years. Domesticated common carp differ from their wild ancestor in morphological, behavioral, physiological, and reproductive traits (Balon, 1995). For example, wild carp has an elongated body with full scale cover, while domesticated carp usually have a much deeper body with four scale patterns: 1) leather, with no scales; 2) line, with large scales along the lateral line; 3) mirror, with a small number of large scattered scales; and 4) fully scaled. Domesticated carp are generally more capable of coping with extreme environments than their wild ancestor (Balon, 1995). Genetic evidence indicates that all common carp populations originate from two ancestral forms of wild carp, the European subspecies (*C. c. carpio*) and the East Asian subspecies (*C. c. haematopterus*) (Chistiakov and Voronova, 2009). The validity of a third subspecies, *C. c. rubrofuscus*, is questionable; it may have diverged from *C. c. haematopterus* (Zhou et al., 2004; Wang et al., 2010; Kohlmann and Kersten, 2013).

During its domestication, common carp has been introduced into many areas. Common carp ancestors have been subjected to genetic interventions, and natural and artificial selection. These factors combined with accumulation of mutations and long-term geographical isolation have produced many varieties of common carp with distinct skin color, body shape, scale pattern, body size, and stress tolerance. Human transport of carp to different geographical locations has generated high levels of gene flow (Wang et al., 2010). Hybrid breeding of carp in China has been common over the last 50 years and has resulted in many varieties or strains, such as Jian carp (JIAN). Multiple rounds of hybridization and genetic introgression were employed during hybrid breeding (Dong et al., 2015). Consequently, the genetic backgrounds of most common carp populations are unknown, especially when breeding history is inadequately recorded or missing. Various genetic tools and molecular markers have been developed and used for studying the phylogenetic relationship among populations and the genetic architecture of populations. These include random amplified polymorphic DNA, amplified fragment length polymorphism, restriction fragment length polymorphism, mitochondrial DNA, and microsatellites (Bartfai et al., 2003; Zhou et al., 2003; Mabuchi et al., 2008; Cheng et al., 2010). However, due to the limited resolution of these genetic markers, many phylogenetic relationships remain uncertain. For example, the origin and relationships among Hebao carp (HB), Xingguo Red carp (XG), Songpu carp (SP), Oujiang color carp (OUJ), and Koi carp (KOI) are controversial (Balon, 1995; Froufe et al., 2002; Wang and Li, 2004a; Wang and Li, 2004b). The previous studies indicated different phylogeny patterns using both nuclear genome (Xu et al., 2014) and mitochondrial genome sequence (Dong et al., 2015; Liu et al., 2019); however, due to limited number of samples, these results were not so solid for validation. Also, the genomic basis of local adaptation shaped by natural selection is still largely unknown. Selective sweep analysis is an effective approach to identify trait-related genes under natural selections or domestications. Xu et al. have reported the selective sweeps in the HB population compared with the SP population and identified *fgfr1a1* in the selective regions (Xu et al., 2014). Another research on Amur ide alkaline adaptation was also conducted using selective sweep method, and dozens of ion transportation-related genes were revealed involving in osmoregulation and pH regulation (Xu et al., 2017). Larger samples would be more persuasive in identification of genes in selective sweep regions due to the high genetic diversity of the populations.

With the fast growth of sequencing technologies, high-throughput genetic markers, such as single-nucleotide polymorphism (SNP), have been used in population genetics. Many studies have demonstrated that SNP arrays can improve the resolution of the differentiation of genetic stocks (Perez-Enriquez et al., 2018; Torati et al., 2019). SNP assays are a useful tool for studying population structure and the effects of natural and artificial selection at the genome scale. For example, the Atlantic salmon SNP array that contained 6,176 informative SNPs was used to genotype 38 anadromous and freshwater wild populations (Bourret et al., 2013). The data illustrated the genetic architecture in salmon and showed the adaptive divergence of SNP allele frequencies across populations and among regional groups. Bradbury et al. applied an SNP array to Atlantic cod and showed an association between SNP allele frequencies and water temperatures across the species range (Bradbury et al., 2011). Jones et al. developed an SNP array to study geographic patterns of genetic variation on stickleback. Substantial genetic variation was found in 34 populations with predominant patterns reflecting demographic history and geographic structure. Genome regions contributing to evolution of marine–freshwater or benthic–limnetic species pairs were identified (Jones et al., 2012).

Many SNP markers have been identified from common carp (Kongchum et al., 2010; Xu et al., 2014), and a high-throughput 250 K common carp SNP array has been developed (Xu et al., 2014). The entire genome sequences of common carp have been published in 2014 (Xu et al., 2014). In the present study, genome-wide SNP genotyping was conducted to determine the genetic architecture of common carp populations and the genetic bases for environmental adaptation. A total of 2198 samples were successfully genotyped with high quality (see Materials and Methods). These samples belonged to 14 different populations, including Yellow River carp (YR), HB, Xingguo carp (XG), OUJ, KOI, Qingshuijiang carp (QSJ), JIAN, Songhe carp (SH), SP, Heilongjiang carp (HLJ), Danube carp (DANU), Szarvas 22 carp (SZ), Tisza carp (TZ), and a population from the USA (AME). YR are mainly cultured along the Yellow River basin of China; HB, XG, and OUJ are mainly cultured in the south of China; SH, SP, and HLJ were mainly cultured in the north of China, and QSJ is mainly cultured on the southwest of China. DANU, SZ, and TZ were collected from Europe, while AME was collected from Alabama in the USA. The phenotypic traits of these common carp populations differ from each other. Most common carp are black or gray, but HB and XG are red. KOI and OUJ have various skin color and patterns, including white, black, red, yellow, blue, and cream. The molecular mechanisms underlying trait differences between different common carp populations were unveiled in this study.

## Materials and Methods

### Sample Collection

The 14 populations of *C. carpio* (2,198 individuals) were randomly collected across Europe, North America, and China. DANU, TZ, and Szarvas 22 (SZ) were collected from the carp live gene bank of the Research Institute for Fisheries, Aquaculture and Irrigation of Hungary (HAKI). North American carp (AME) were collected from the Chattahoochee River in Alabama in the USA. Ten other populations were sampled from China, namely, the YR from Zhengzhou of Henan Province, HB from Wuyuan of Jiangxi Province, XG from Xingguo of Jiangxi Province, OUJ from Oujiang of Zhejiang Province, KOI from Beijing, QSJ from Guiyang of Guizhou Province, JIAN from Wuxi of Jiangsu Province, SH and SP from Harbin of Heilongjiang Province, and HLJ from Mudanjiang of Heilongjiang Province. The numbers of samples from each population are shown in **Table S1**.

### DNA Extraction, Genotyping, and Quality Control

Genomic DNA was extracted from blood or fin samples using a DNeasy 96 Blood & Tissue Kit (Qiagen, Shanghai, China) following the manufacturer’s protocol. Extracted DNA was quantified by a Nanodrop-1000 spectrophotometer (Thermo Scientific, Wilmington, DE, USA). DNA integrity was examined on a 1.0% agarose gel by electrophoresis. The final DNA concentration was diluted to 50 ng/μl for genotyping with an amount of 2 μg per sample. The common carp 250-K SNP array was developed using Affymetrix Axiom genotyping technology. Genotyping was performed by GeneSeek (Lincoln, Nebraska, USA). After genotyping, PLINK v1.9 software (https://www.cog-genomics.org/plink2) was used for quality control (Chang et al., 2015). SNPs with low call rate (< 95%) or low minor allele frequency (MAF < 5%) were excluded, and samples with <90% genotyping rate were filtered out. The filtered genotype file was uploaded into the European Nucleotide Archive database (https://www.ebi.ac.uk/ena/data/view/PRJEB33066).

### Phylogeny, Principal Component Analysis, and Genetic Structure

A maximum-likelihood tree was constructed by RAxML with 1,000 bootstraps (Stamatakis, 2014), and the tree was displayed with iTOL software (http://itol.embl.de/upload.cgi). Principal component analysis (PCA) was conducted using GATK software (Van der Auwera et al., 2013), and all of the SNPs were used to investigate the population structure using Structure 2.3.1 software with 2,000 iterations and the MCMC model (Falush et al., 2007). The optimal *K* value was selected by Delta *K* method (Evanno et al., 2005). The resulting structure matrix was plotted using StructurePlot 2.0 software (Ramasamy et al., 2014).

### LD Decay and Haplotype Construction

Linkage disequilibrium (LD) decays for the main populations and all of the samples were calculated within a range of 50 kb using PLINK (Chang et al., 2015). The average *R* ^2^ value of each 1 kb region was calculated (Zhou et al., 2016; Xu et al., 2017). All of the *R* ^2^ values were then plotted against the physical distances of SNPs in units of kb. Haplotype blocks in different populations were identified by PLINK software using the “--blocks” parameter.

### Calculation of π Ratio, Fst, Tajima’s *D*, and Identification of Selective Signatures

We calculated the π distribution for each linkage group using a sliding window method in Vcftools. The window width was set to 100 kb, and the stepwise distance was 100 kb. The π values from the main populations were compared, and the ratios were sorted. Fst and Tajima’s *D* values were also calculated using Vcftools with the parameters “–weir-fst-pop” and “–TajimaD,” respectively. We identified the regions with the 5% highest π ratios and the regions with the 5% highest Fst values. Together with regions identified on the basis of the above two thresholds, genes within selective sweeps were annotated using GOEAST for Gene Ontology (GO) (Young et al., 2010) and the DAVID software (Dennis et al., 2003) for KEGG pathway analysis. Scatter plots of π and Fst values were generated using the ggplot2 package of the Comprehensive R Archive Network (http://cran.r-project.org/package=ggplot2).

## Results and Discussion

### Sample Collection and Genotyping

**Figure 1:**
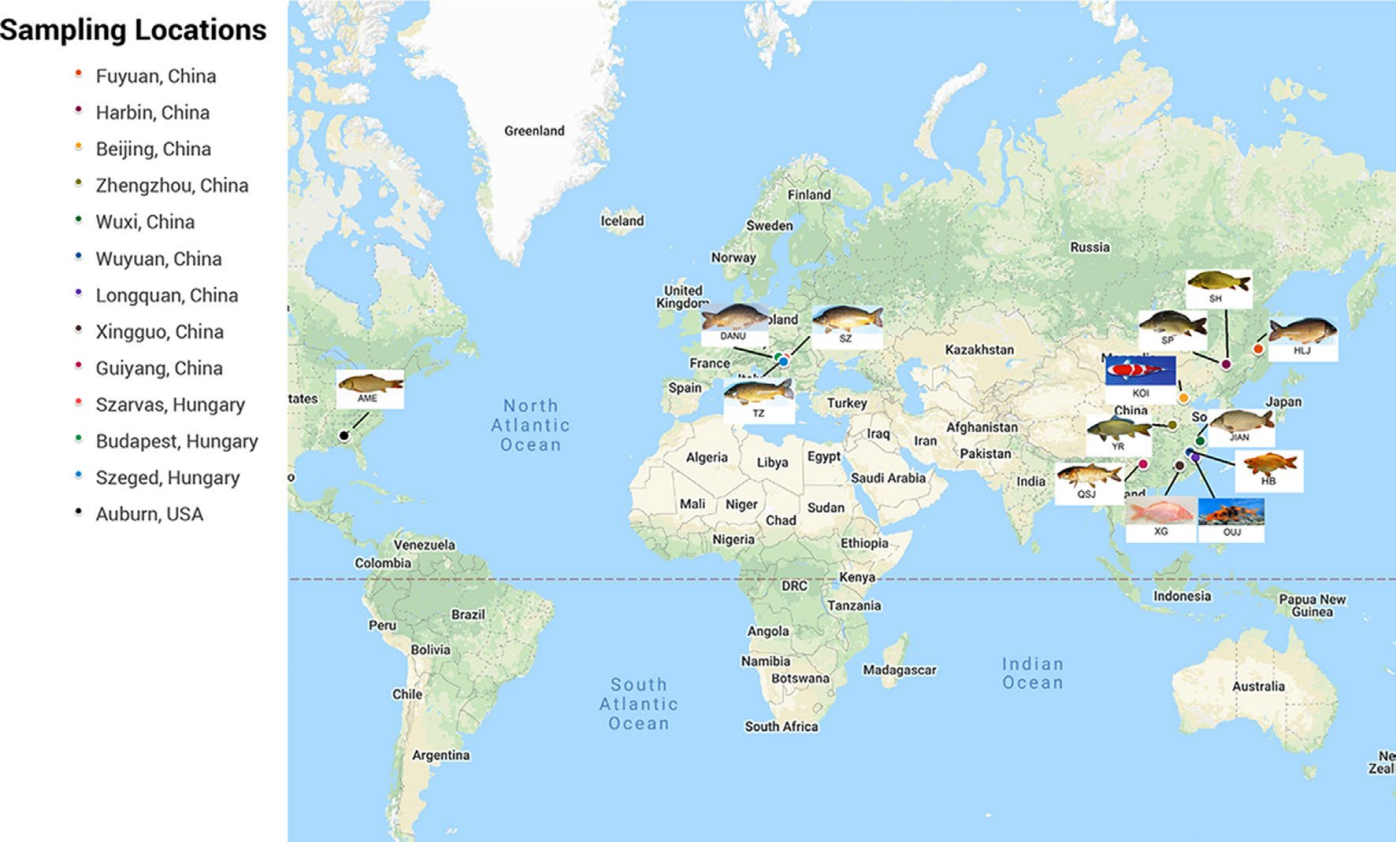
Sampling locations of 14 populations of *C. carpio*.

A total of 14 populations of *C. carpio* were collected from 13 locations in China, Hungary, and the USA (**Figure 1**). In addition to the geographic divergences of these populations, several populations were included due to their special biological features, such as scale pattern (SP population), red body color (HB population, XG population, and QH group in the OUJ population), and purse-like body shape (HB population). After DNA extraction and SNP genotyping using Carp 250-K SNP array, a raw genotype database of 222,694 SNPs for 2,198 samples was generated. A total of 2,198 samples with 134,719 polymorphic SNPs passed the quality control threshold and were used for further analysis. Sample information for each population is shown in **Table S1**.

### Phylogeny, PCA, and Population Structure

**Figure 2:**
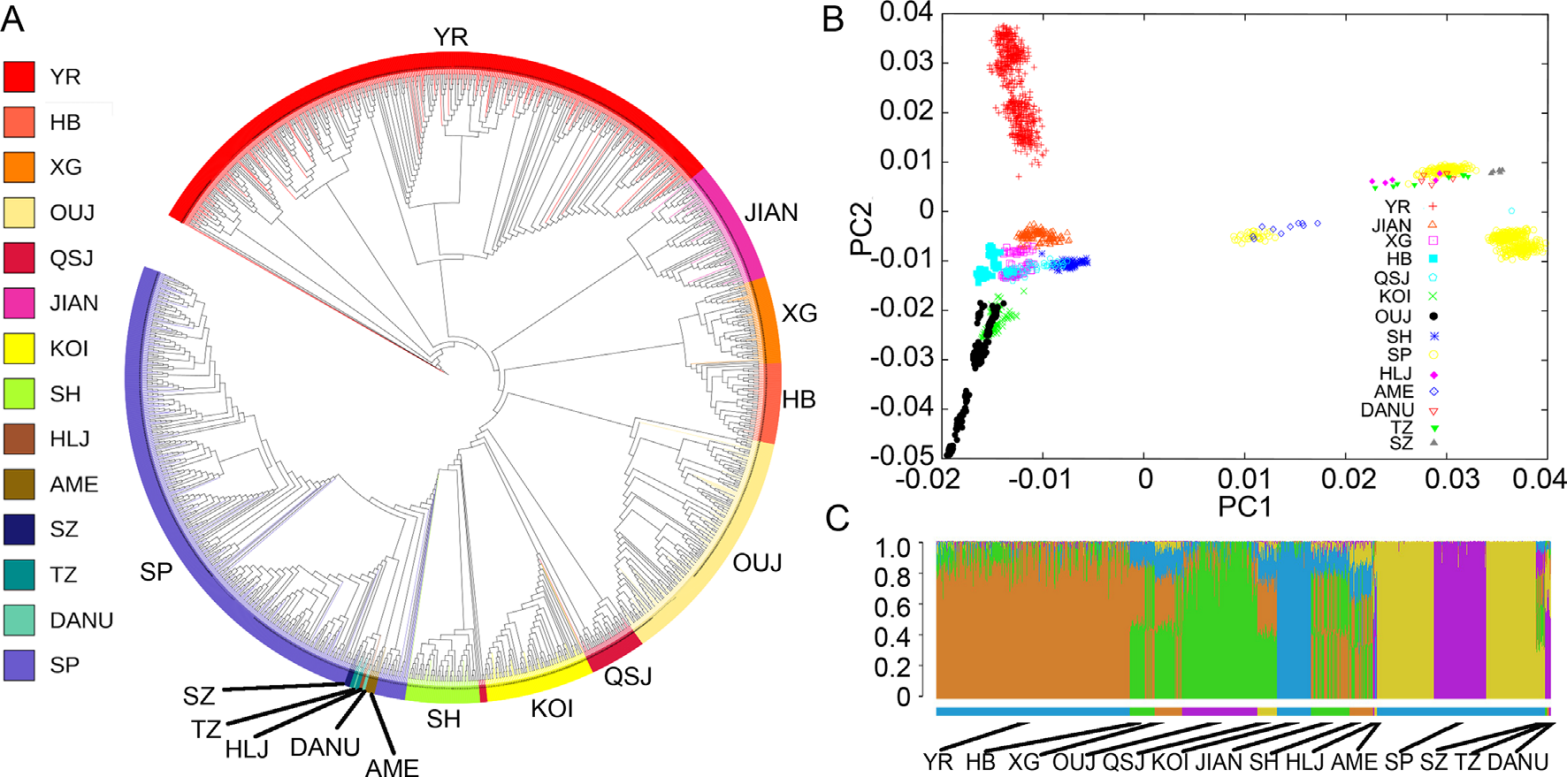
Phylogeny, principal component analysis (PCA), and genetic structure of 14 populations of *C. carpio*. **(A)** A maximum-likelihood phylogenetic tree of 14 populations of common carp generated on the basis of polymorphic single-nucleotide polymorphisms (SNPs). Population abbreviations: SP, Songpu; DANU, Danube; SZ, Szarvas; TZ, Tisza; AME, North American; YR, Yellow River; HLJ, Heilongjiang; OUJ, Oujiang color; HB, Hebao; XG, Xingguo; KOI, Koi; SH, Songhe; JIAN, Jian; QSJ, Qingshuijiang. **(B)** PCA of *C. carpio* populations. **(C)** The population structure of common carp populations. Each color represents one ancestral population; each individual is represented by a vertical bar, and the length of each colored segment in each vertical bar represents the proportion contributed by ancestral populations. *K* = 5 was used for analysis with the highest Delta *K* value.

To investigate the divergence of the representative *C. carpio* populations from different locations, we constructed the phylogenetic tree using the whole genotyping data (**Figure 2A**). The Asian and European populations formed two distinct clades, while the AME population grouped with the European clade. The SP population constituted the major part of the European clade as it was bred from mirror carp originally introduced from Europe in the 1950s. A similar result was indicated by PCA, showing subgroups in either Asian populations or European populations (**Figure 2B**). In the Asian populations, YR formed a tight cluster and other populations (JIAN, HB, XG, KOI, OUJ, and QSJ) formed another subgroup. Three subgroups could be identified in the European cluster. The first subgroup contained only SP samples which were closely grouped. The second subgroup included three Hungary populations (DANU, TZ, and SZ), SP population, and HLJ population, indicating close relationship among SP, HLJ, and Hungary carp. A small number of SP samples and all of the AME samples formed the third subgroup, showing that the common carp in the USA might have originated from European populations. We analyzed the population structure using the Bayesian clustering program STRUCTURE. Since the values of Delta *K* from ln likelihood were high for the models *K* = 5, we showed the clusters of *K* = 5 in **Figure 2C**. The Asian populations were separated into two subgroups, similar to the PCA result, and the European populations showed shared common ancestry. Within the Asian populations, obvious genetic admixture was observed in the YR population and other populations, except for KOI and OUJ. The KOI population has had a highly inbred history to maintain the purity of the genetic component, and the OUJ population also showed relatively pure genetic structure due to their habitats in the isolated mountainous areas in Zhejiang, China. This result is in accordance with our previous study (Xu et al., 2014), but there were slight differences in the shared genetic components in all of the populations.

### LD Decay and Haplotype Construction

**Figure 3:**
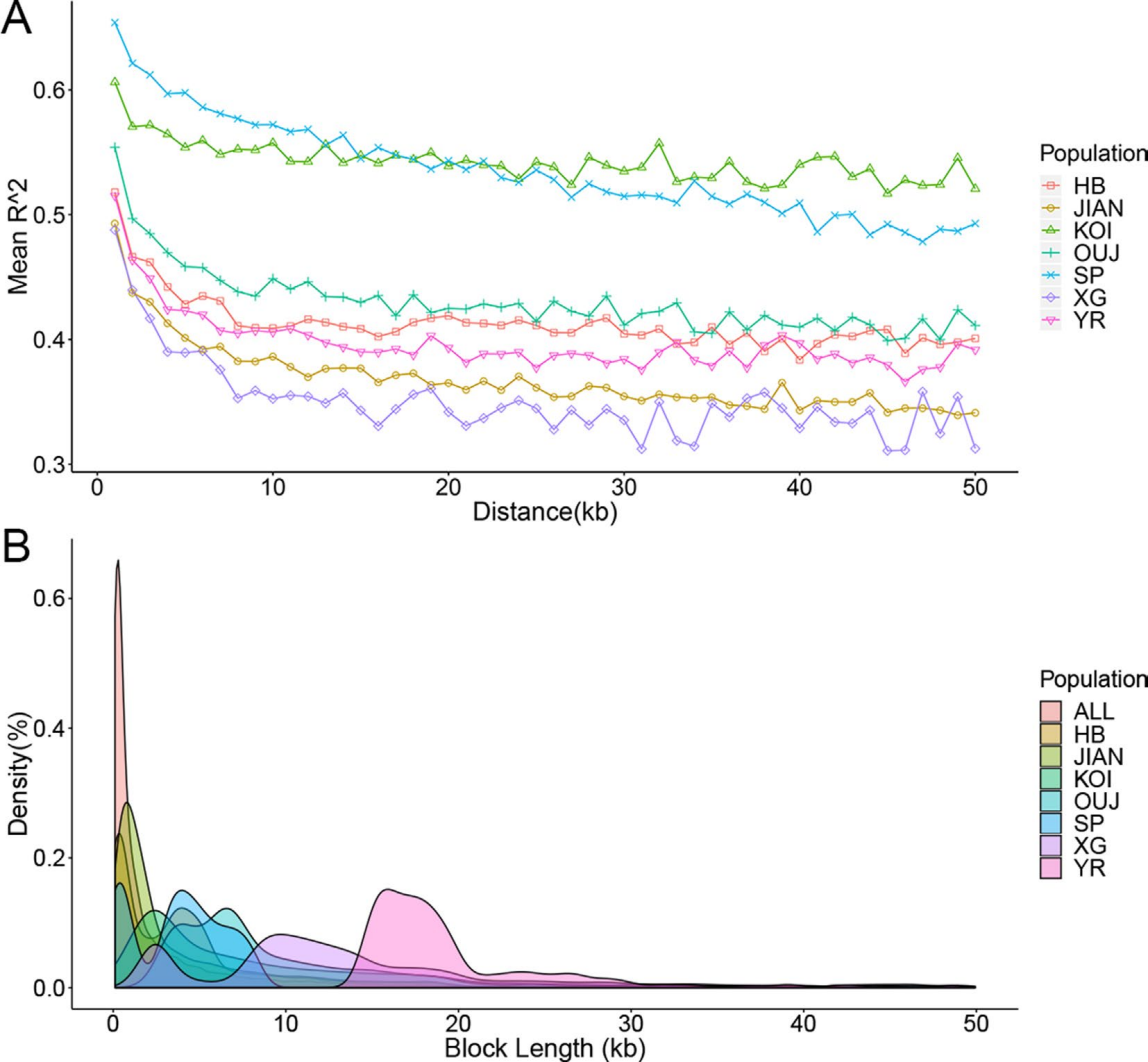
Linkage disequilibrium (LD) decay and haplotype distribution. **(A)** LD decay of seven populations. The *X*-axis represents the distances (kb) between paired SNPs, and the *Y*-axis represents mean *R* ^2^ of the SNP pairs within each distance region. **(B)** Haplotype distribution of all of the samples and seven populations. The *X*-axis represents the lengths (kb) of haplotype blocks, and the *Y*-axis represents the density (percentage in all of the blocks) of each block with certain lengths.

Based on the genome assembly and SNP array for *C. carpio*, the LD was investigated for seven main populations that contained an adequate number of samples. The *R* ^2^ value among each pair of SNPs was calculated using PLINK software, and the raw data were classified by distance ranges. The LD decays with the extension of the distance between SNPs and different populations showed distinct LD decay patterns (**Figure 3A**). KOI and OUJ had significantly higher *R* ^2^ values than five other populations, which was consistent with the population structure results. Haplotypes were constructed for all samples and seven populations using PLINK. The distribution of different haplotype lengths was calculated by ggplot2 package, and OUJ, SP, XG, and YR showed longer blocks (**Figure 3B** and **Table S2**). Haplotypes are useful in GWAS analysis and provide more SNP genotyping information through imputation.

### Genome-wide Selective Sweep Analysis

*C. carpio* is a genetically diverse species that has adapted to a variety of environments in Eurasia and has been domesticated for more than 2,000 years (Xu et al., 2014). *C. carpio* has been bred into numerous strains, generating distinct phenotypes in body color, scale pattern, and body shape. These characters are partially attributable to genome diversity due to environmental adaptation.

**Figure 4:**
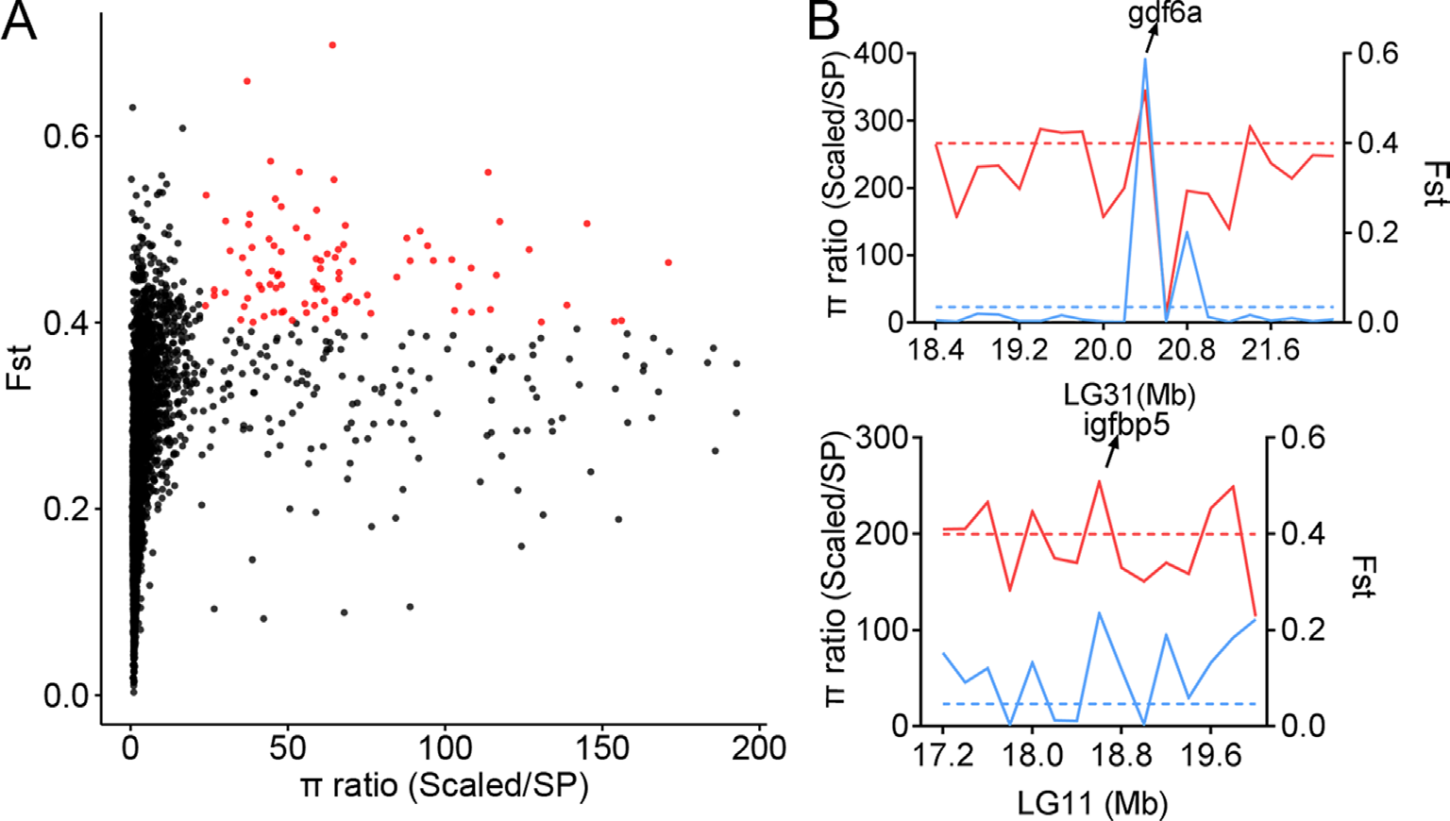
Selective sweeps and genetic diversity of selected genes. **(A)** Distribution of π and Fst values in the comparison between scaled and SP populations. The *X*-axis represents the π ratio values of all of the sliding windows, and the *Y*-axis represents the Fst values of all of the sliding windows. Red dots represent windows that passed thresholds of both π and Fst. **(B)** π ratio and Fst values within windows neighboring selected genes. The solid blue line represents the π ratio, and the dashed blue line represents the π ratio threshold. The solid red line represents the Fst, and the dashed red line represents the Fst threshold.

The genetic diversity in certain genome regions might be reduced due to natural selection. To identify the genome regions under selective pressure in populations with distinct biological features, we scanned the genome-wide variations and allele frequency spectra of the 134,719 SNPs. The areas of comparison included scales, body shape, and body color. The π ratios of three groups (πScaled carp/SP, πYR/HB, and πQH/FY) were calculated using a 200-kb sliding-window approach with Vcftools software. In comparison to the Scaled carp in Asia (including YR, HB, XG, OUJ, QSJ, KOI, and JIAN), the SP population had distinct genetic diversities across the whole genome (**Figure 4A**). We identified 321 significant windows corresponding to 64.2 Mb in size (top 5%, empirical π ratios ≥ 23.17), which included 289 candidate genes based on the π ratio analysis. To validate the genome regions under strong selective sweeps in the SP population, the genome regions with Fst greater than 0.3995 (top 5%) were also identified, corresponding to 64.2 Mb and 285 candidate genes. A total of 100 candidate genes shared by both the π ratio and Fst analysis were identified as potentially affected genes under selective sweeps (**Figure 4A**, **Table S3**).

The results suggest that the genomes of the SP population have been significantly altered, by the environment, into a no-scale pattern. Fish scale is an epidermal appendage and it is an important protective tissue. Among the samples, all were fully scaled except for the SP carp that lacks scales. Through selection sweeping analysis of comparing SP carp with other fully scaled carps, the significant genome regions were identified (**Table S3**). Also, several target genes were found that might be related to the scale pattern (**Figure 4B**). Growth differentiation factor 6a (*gdf6a*), also named cartilage derived morphogenetic protein 2, is a member of the BMP family. The expression of the *gdf6a* gene has been detected in both fetal and post-natal cartilaginous tissues involved in the development of long bones. The *gdf6a* gene may play a role in suppression of ossification (Wei et al., 2016). Insulin-like growth factor-binding protein 5 (*igfbp5*) is a member of the IGFBP family that can either inhibit or stimulate the growth-promoting effects of the IGFs on cell culture. It is involved in the Ras/p38 MAPK signaling pathway in regulating cell proliferation and apoptosis (Yang et al., 2018). To study these candidate genes and their potential functions, GO and KEGG analyses were performed on the candidate genes, offering insight into the genetic evolution and adaptive mechanisms of the SP population (**Tables S4** and **S9**).

We also investigated selective sweeps in comparisons (YR vs HB, QH vs FY), which showed distinct body shapes or body color, respectively. YR and HB were compared because they represent the typical populations in central China and southern China, respectively. QH and FY were compared due to their high similarity in genome background and the possibility that it might be easier to screen out potential genes associated with body color. We identified a total of 321 significant windows corresponding to 64.2 Mb in size for each comparison (top 5%, empirical π ratios ≥ 1.3121, 1.9341, respectively), which included 293 and 287 candidate genes, respectively. To further validate the genome regions under strong selective sweeps in the HB or FY population, the genome regions with Fst greater than 0.2588 or 0.1622 (top 5%) were also identified, including 278 and 292 candidate genes, respectively. A total of 38 and 65 candidate genes shared by both the π ratio and Fst analysis were recognized, respectively, as genes potentially affected under selective sweeps (**Figures S1A** and **S2A**, **Tables S5** and **S7**).

The purse-like shape of the HB population was probably due to extensive growth of muscle or bones compared to the YR population, and several genes (*trhr* and *bmpr1b*) were screened out for their potential functions in bone and muscle development (**Figure S1B**). Previous genome-wide association and replication studies identified *trhr* as a gene associated with lean body mass (Liu et al., 2009). The gene *bmpr1b* is engaged in the regulation of skeletal development through interactions with FGFR families (Qi et al., 2014). GO enrichment analysis was also performed on the candidate genes, offering insight into the genetic evolution and adaptive mechanisms of the HB population (**Tables S6** and **S9**). GO terms including thyrotropin-releasing hormone receptor activity, Wnt-activated receptor activity, and transforming growth factor beta-activated receptor activity were enriched, providing clues for more detailed analysis. Coloration is an important trait for common carp, especially for ornamental strains, since it is often a criterion for visually determining quality and market value. OUJ carp is a famous ornamental farmed fish, which has four distinct color patterns, namely, whole white (FY), whole red (QH), white with scattered big black spots, and red with scattered big black spots. FY and QH were compared, and several potential target genes were identified (**Figure S2B**). The keratinocyte growth factor (fgf7/kgf) can promote melanosome transfer and act on recipient keratinocytes through stimulation of the phagocytic process. Fgf7 affects keratinocytes derived from different skin color (Cardinali et al., 2008). Another gene in selective sweep regions, *opsin5*, has been reported engaging in phototransduction and regulates seasonal changes in color perception (Shimmura et al., 2017). It was identified as an ultraviolet (UV)-sensitive pigment of the retina and other photosensitive organs in birds (Ohuchi et al., 2012). GO and KEGG analyses were also performed on the candidate genes and offered insight into the genetic evolution and adaptive mechanisms of the SP population (**Tables S8** and **S9**). Several significant pathways were enriched, including dopamine receptor signaling pathway and dopamine neurotransmitter receptor activity. This indicated the importance of dopamine-related networks in body color determination. The representative GO terms and pathways enriched in these comparisons were selected, and the Fst values of genes in these pathways were significantly higher than the whole-genome level (**Figure S3**).

## Conclusions

We investigated the genomic divergence among various populations of *C. carpio*. Distinct genetic component differences were identified between Asian and European populations. The haplotypes of each population could benefit research on trait associations. Selective sweep analyses results showed that hundreds of genes within selective sweep regions were identified by genome scanning among different populations, including *gdf6a*, *bmpr1b*, and *opsin5*. This study comprehensively revealed genetic structure of global populations of *C. carpio*, and potential trait-related genes could be valuable for genome-assisted breeding of *C. carpio*.

## Ethics Statement

This study was carried out in accordance with the recommendations of the care and use of animals for scientific purposes set up by the Animal Care and Use Committee of Chinese Academy of Fishery Sciences (ACUC-CAFS). The protocol was approved by the ACUC-CAFS. Before the blood or fin samples were collected, all of the fishes were euthanized in MS222 solution.

## Author Contributions

PX initiated and coordinated the research project. JX and YJ conducted the analysis and drafted the manuscript. ZZ, HZ, and JF engaged in sample collection and genotyping analysis. CD, WP, BC, and RT took part in enrichment analysis. All authors read and approved the final manuscript.

## Funding

This work was supported by Central Public-Interest Scientific Institution Basal Research Fund, CAFS (No. 2016GH02, No. 2016HY-JC0301, and No. 2016HY-ZD0302), the National Natural Science Foundation of China (No. 31502151 and No. 31422057), the National High-Technology Research and Development Program of China (2011AA100401), the National Key Research and Development Program (2018YFD0900102), and the National Infrastructure of Fishery Germplasm Resources of China (No. 2018DKA30470).

## Conflict of Interest Statement

The authors declare that the research was conducted in the absence of any commercial or financial relationships that could be construed as a potential conflict of interest.

The handling editor is currently editing co-organizing a Research Topic with one of the authors PX, and confirms the absence of any other collaboration.

## Supplementary Material

Supplementary Tables 1–9 Summary of genotyping data, haplotype blocks, and selective sweep results.

Supplementary Figure 1 π, Fst distribution, and selected genes in selective sweep analysis between YR and HB populations. (A) Distribution of π and Fst values in the comparison between YR and HB populations. The *X*-axis represents the π ratio values of all sliding windows, and the *Y*-axis represents the Fst values of all sliding-windows. Red dots represent windows that passed thresholds of both π and Fst. (B) π ratio and Fst values within windows neighboring selected genes. The solid blue line represents the π ratio, and the dashed blue line represents the π ratio threshold. The solid red line represents the Fst, and the dashed red line represents the Fst threshold.

Supplementary Figure 2 π, Fst distribution, and selected genes in selective sweep analysis between QH and FY samples. (A) Distribution of π and Fst values in the comparison between QH and FY samples. The *X*-axis represents the π ratio values of all sliding windows, and the *Y*-axis represents the Fst values of all sliding windows. Red dots represent windows that passed thresholds of both π and Fst. (B) π ratio and Fst values within windows neighboring selected genes. The solid blue line represents the π ratio, and the dashed blue line represents the π ratio threshold. The solid red line represents the Fst, and the dashed red line represents the Fst threshold.

Supplementary Figure 3 Box plot of the Fst differences between representative GO terms and the whole genome. Boxes denote the values between the 25th and 75th percentiles and the black transverse line inside the box denotes the median. Black vertical lines denote the values within 1.5 times of quartile values. Outliers are shown as black dots. (A) Scaled vs SP. (B) YR vs HB. (C) QH vs FY.
