# Supplementary figures and images for: Patterns of Geographical and Potential Adaptive Divergence in the Genome of the Common Carp (Cyprinus carpio)
Source: Front Genet. 2019 Jul 12;10:660. doi: 10.3389/fgene.2019.00660 (PMC6640160; doi:10.3389/fgene.2019.00660)

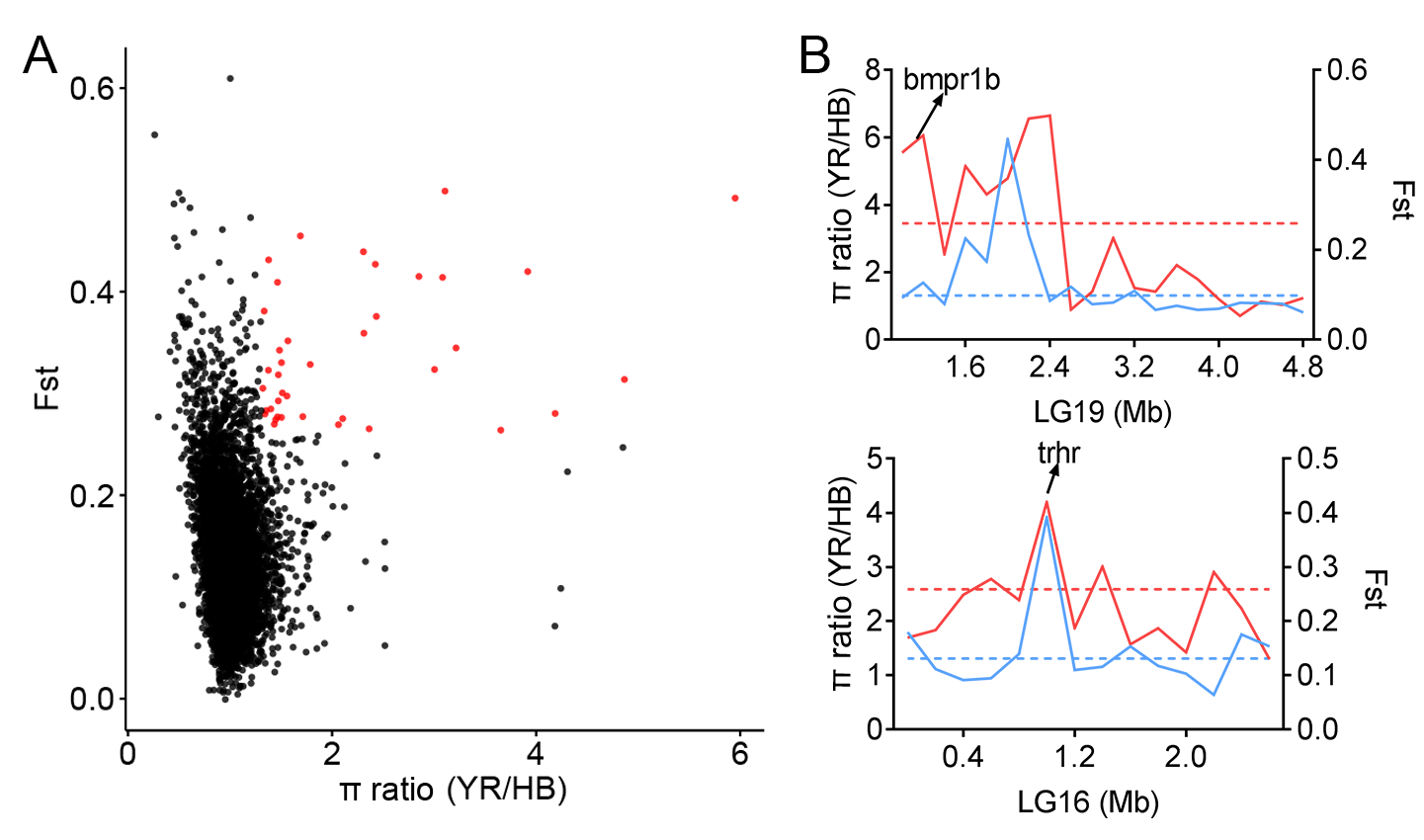

Supplement: Supplementary Figure 1 — π, Fst distribution, and selected genes in selective sweep analysis between YR and HB populations. (A) Distribution of π and Fst values in the comparison between YR and HB populations. The X-axis represents the π ratio values of all sliding windows, and the Y-axis represents the Fst values of all sliding-windows. Red dots represent windows that passed thresholds of both π and Fst. (B) π ratio and Fst values within windows neighboring selected genes. The solid blue line represents the π ratio, and the dashed blue line represents the π ratio threshold. The solid red line represents the Fst, and the dashed red line represents the Fst threshold. [file Image_1.tif]

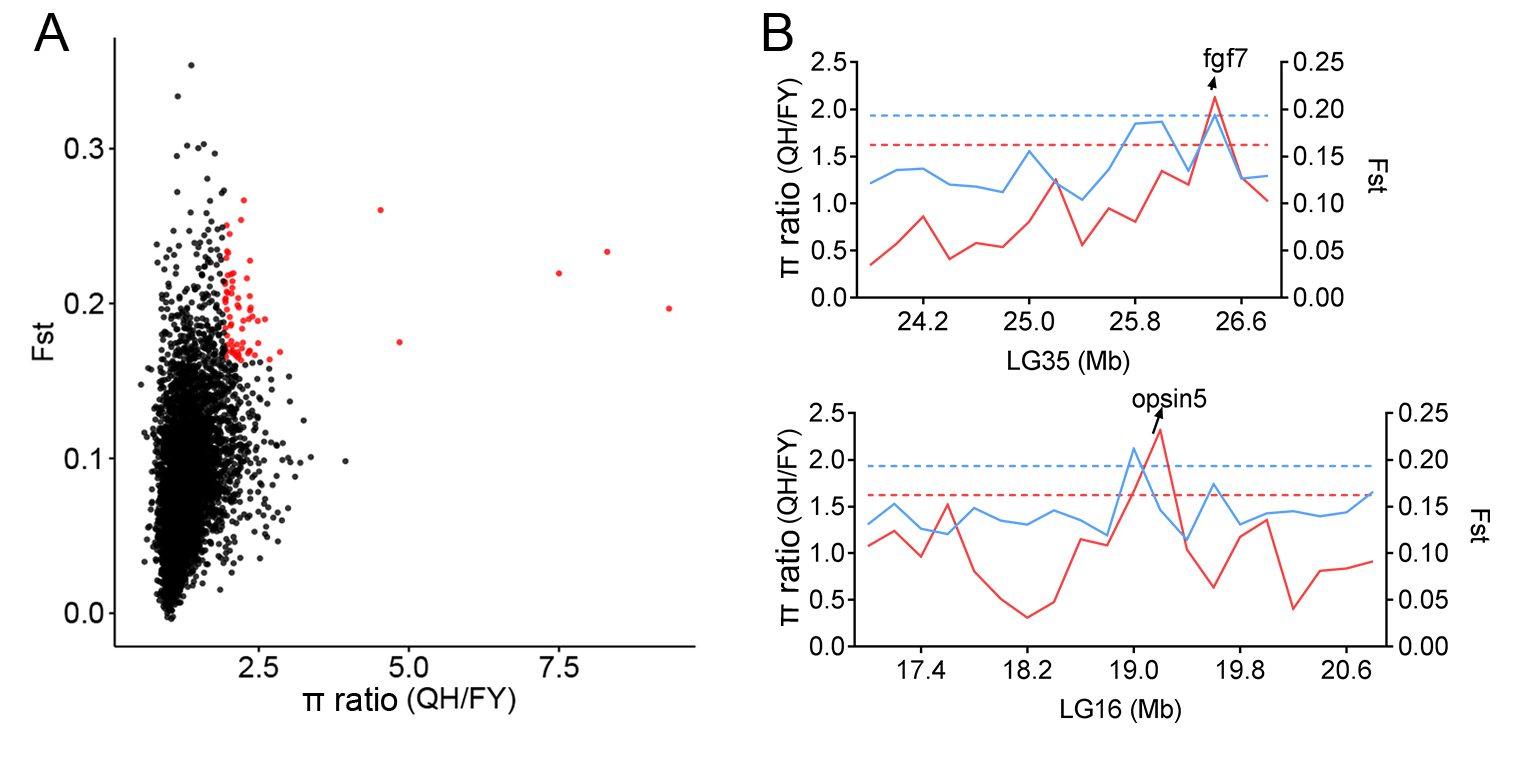

Supplement: Supplementary Figure 2 — π, Fst distribution, and selected genes in selective sweep analysis between QH and FY samples. (A) Distribution of π and Fst values in the comparison between QH and FY samples. The X-axis represents the π ratio values of all sliding windows, and the Y-axis represents the Fst values of all sliding windows. Red dots represent windows that passed thresholds of both π and Fst. (B) π ratio and Fst values within windows neighboring selected genes. The solid blue line represents the π ratio, and the dashed blue line represents the π ratio threshold. The solid red line represents the Fst, and the dashed red line represents the Fst threshold. [file Image_2.tif]

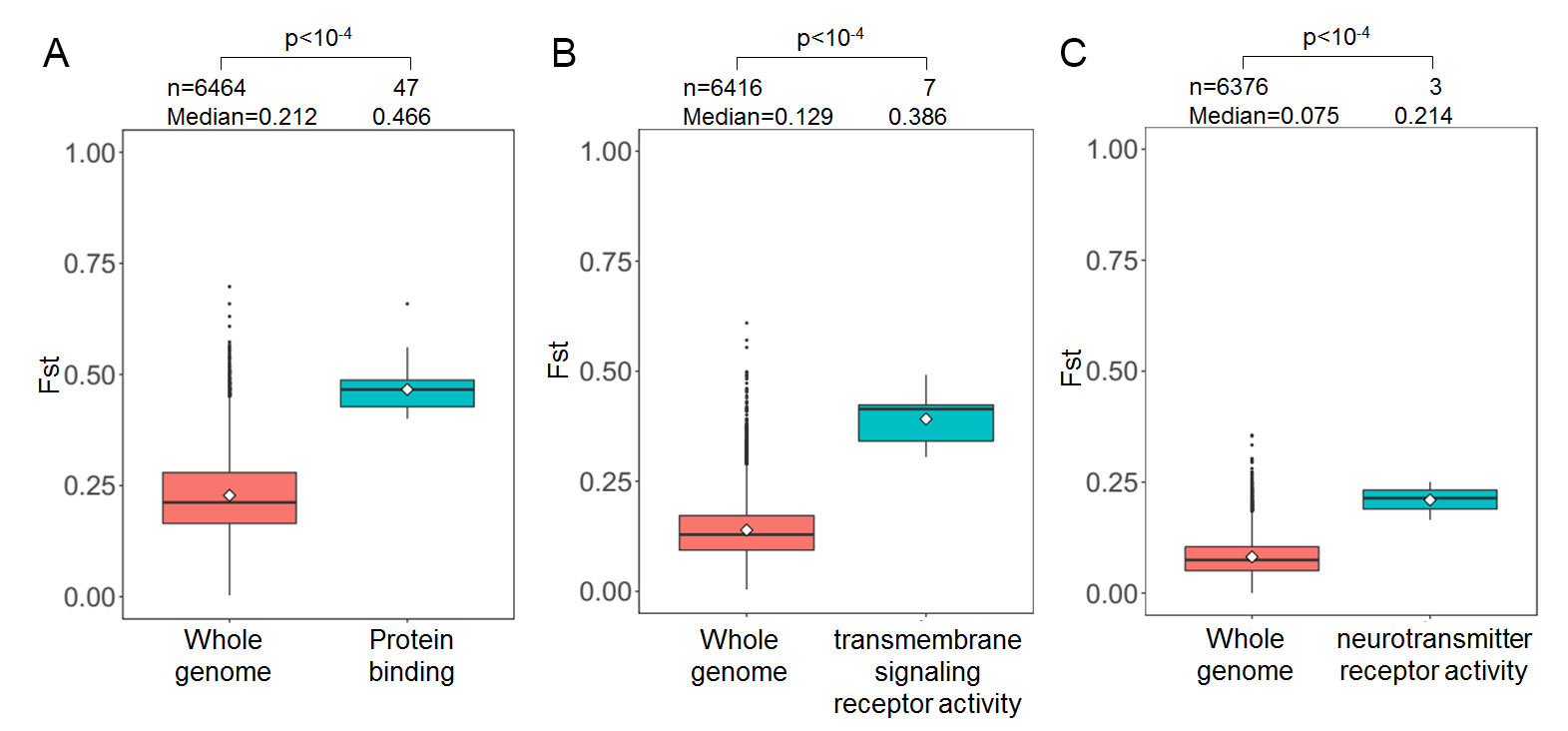

Supplement: Supplementary Figure 3 — Box plot of the Fst differences between representative GO terms and the whole genome. Boxes denote the values between the 25th and 75th percentiles and the black transverse line inside the box denotes the median. Black vertical lines denote the values within 1.5 times of quartile values. Outliers are shown as black dots. (A) Scaled vs SP. (B) YR vs HB. (C) QH vs FY. [file Image_3.tif]
